# ViralFusionSeq: accurately discover viral integration events and reconstruct fusion transcripts at single-base resolution

**DOI:** 10.1093/bioinformatics/btt011

**Published:** 2013-01-12

**Authors:** Jing-Woei Li, Raymond Wan, Chi-Shing Yu, Ngai Na Co, Nathalie Wong, Ting-Fung Chan

**Affiliations:** ^1^School of Life Sciences, ^2^Hong Kong Bioinformatics Centre and ^3^Department of Anatomical and Cellular Pathology, The Chinese University of Hong Kong, Shatin, Hong Kong

## Abstract

**Summary:** Insertional mutagenesis from virus infection is an important pathogenic risk for the development of cancer. Despite the advent of high-throughput sequencing, discovery of viral integration sites and expressed viral fusion events are still limited. Here, we present ViralFusionSeq (VFS), which combines soft-clipping information, read-pair analysis and targeted *de novo* assembly to discover and annotate viral–human fusions. VFS was used in an RNA-Seq experiment, simulated DNA-Seq experiment and re-analysis of published DNA-Seq datasets. Our experiments demonstrated that VFS is both sensitive and highly accurate.

**Availability**: VFS is distributed under GPL version 3 at http://hkbic.cuhk.edu.hk/software/viralfusionseq

**Contact**: tf.chan@cuhk.edu.hk

**Supplementary information**: Supplementary data are available at *Bioinformatics* Online

## 1 INTRODUCTION

Viral infection accounts for 15–20% of human cancers (Morissette and Flamand, 2010). Well-known cancer-associated viruses include the Human papillomavirus (HPV) and the Epstein–Barr virus (EBV), which are present in nearly all cervical cancers (Walboomers *et al.*, 1999) and nasopharyngeal carcinoma (NPC) (Young and Rickinson, 2004), respectively. Hepatitis B virus (HBV) infection is a strong etiologic factor for hepatocellular carcinoma worldwide (Chemin and Zoulim, 2009). Some viruses like HPV and HBV commonly integrate into the host genome, where they predispose to genome instability and cancer risks (Zhao *et al.*, 2012). Nevertheless, the ability to precisely locate the viral insertional sites has long been hindered by previous low-resolution techniques and thus limiting research into the mutagenic effects of such integrations. Viral integration in the host can either be episomal or induce viral–human fusion transcript (Schmitz *et al.*, 2012). Integration of HBV can be detected in as much as 90% of hepatocellular carcinoma (HCC), where clonal expansion of the same integration site has been reported (Cougot *et al.*, 2005; Jiang *et al.*, 2012; Sung *et al.*, 2012). It is hence not only important to identify the sites of genome integrations, but also to discover transcribed viral–human sequences, which both may possess a functional role in tumorigenesis. SeqMap 2.0, an earlier web-based system, uses pre-defined viral features to locate viral integration sites (Hawkins *et al.*, 2011). Unfortunately, their framework is specific to the 454 sequencing platform and does not address the concerns many have of data privacy. Besides, reliability of the putative fusion breakpoints was not evaluated. More importantly, as HBV has no preferential sites in the human genome to be integrated into (Kraus *et al.*, 2008; Wentzensen *et al.*, 2002), the framework could not discover novel viral–human integrations. More recently, VirusSeq was proposed for detecting the presence of viral species in sequence data, and finding viral integration events using discordant Read Pair (RP) information. Through alignments, VirusSeq was able to identify regions of a chromosome that fused with a virus (Chen *et al.*, 2013).

Here, we propose a genome-wide viral fusion discovery and annotation pipeline. Our method resembles CREST (Wang *et al.*, 2011) and ClipCrop (Suzuki *et al.*, 2011), both of which use soft-clipping to identify genomic structural variations. What sets our method apart from theirs is our focus on viral integration and the use of viral genome(s) as the primary input to our pipeline. Our unified pipeline ViralFusionSeq (VFS) is used for discovering viral integration events and expressed fusion transcripts using high-throughput sequencing (HTS). The most notable difference between VFS and other tools is that VFS uses both RP and Clipped Sequence (CS) information to find viral fusion events and breakpoints (Supplementary Section S1). Using the latter, VFS is able to discern fusion breakpoints accurately to single-base resolution. Moreover, VFS is generalized to major sequencing platforms, and is applicable to both DNA- and RNA-Seq data.

## 2 METHODS

### 2.1 RNA-Seq, simulation experiment and re-analysis of real DNA-Seq data

We performed paired-end transcriptomic sequencing on a HBV-infected HCC cell-line *HKCI-5a* by Illumina HiSeq 2000 (Supplementary Section S2). We applied VFS on this RNA-Seq data, followed by validation with Sanger sequencing. Afterwards, we demonstrated VFS on our simulated DNA-Seq dataset and re-analyzed a published DNA dataset (Sung *et al.*, 2012).

### 2.2 Discovery of putative fusion events

Using the Burrows-Wheeler Aligner (BWA) (Li and Durbin, 2009), VFS starts with pre-processing the sequence reads according to BWA’s trimming algorithm. Quality-trimmed sequence reads are then mapped onto viral sequences. Sensitive mapping is achieved by the use of (**i**) viral and human decoy sequences that have incorporated different haplotypes or assemblies of references to allow mapping of reads originated from rather divergent strains or sequenced subjects, and (**ii**) the use of BWA-SW algorithm implemented in BWA (Li and Durbin, 2010), which is optimized for the increasingly common longer sequencing reads.

**Fig. 1.**
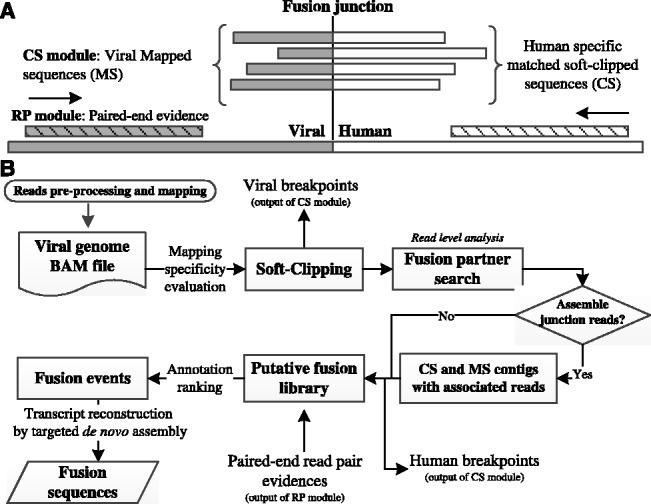
(**A**) Schematic of reads alignment. Fusion breakpoints between viral (grey) and human (white) sequences are identified by soft-clipped alignment. Paired-end reads (diagonal) substantiate the fusion event and assist in transcript reconstruction. (**B**) Overview of VFS

BWA-SW performs Smith–Waterman local alignment. For viral–human chimeric sequence reads, the viral portion would be aligned as mapped sequences (MS), leaving the unaligned human CS as overhang. These overhangs are soft-clipped and the sequence is retained in the alignment file (Li *et al.*, 2009). VFS extracts all CS and MS and determines breakpoints using the soft-clipping information. Specificity of mapping to viral sequence is evaluated to avoid false mapping, which might happen due to simultaneously mapping an excessive quantity of sequence reads onto viral sequences. The function, implemented by BLAST, scrutinizes both the MS and CS for significant matches to non-target species. In the process of identifying fusion partners, read-level analysis is performed (Fig. 1).

### 2.3 Reliability of fusion breakpoint and annotation ranking

VFS uses a simple yet effective empirical statistical method to evaluate the quality of fusion breakpoint and rank fusion’s annotation. The concept is based on the Minimal Match on Either side of Fusion (**MMEF**) (Wang *et al.*, 2010). For each fusion event, the reliability is directly computed by MMEF using the following equation:

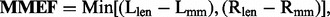

where L_len_ and R_len_ represent alignment lengths and L_mn_ and R_mn_ indicate mismatches along the alignment. The sub-score of each fusion partner is directly calculated by subtracting *len* by *mm*. The best fusion candidate with the highest sub-score is selected. The composite MMEF score is the minimal of the two sub-scores. The score becomes higher when the sequence length of the respective side of the fusion is more balanced, conferring higher uniqueness to the respective genome or gene-set of the target species.

Fusion events are annotated by numerous data sources, including NCBI *Nucleotide and RefSeq databases* and human repetitive elements identified by RepeatMasker, which were obtained from the UCSC Genome Browser.

### 2.4 Reconstruction of fusion transcript by paired-end information

VFS is capable of exploiting both CS and RP information to reconstruct fusion transcripts. Fusion breakpoint sequences are used as seeds to perform targeted assembly on RNA-Seq data. VFS executes the RP fusion detection method to identify all sequence reads with one end mapped onto the viral genome and the other on to the human genome. Then, sequences mapped onto respective genomes are subjected to targeted *de novo* assembly; these include (i) CS and their paired mates from the CS module; (ii) reads from the RP module; and (iii) RPs with one end mapped in the vicinity (500 bp) of the human regions reported by the RP module.

## 3 RESULTS AND DISCUSSION

### 3.1 RNA-Seq experiment

Viral integration in HCC often elicits transcriptional impact on cancer marker genes, suggesting the importance of expressed fusion transcripts (Jiang *et al.*, 2012). We performed RNA-Seq on *HKCI-5* to a depth of 11 Gb. VFS identified three candidate fusion events in *HKCI-5a*, of which all could be successfully validated by Sanger sequencing. We highlight the most complicated fusion transcript formed between the HBV core gene and the human chr7 containing *CDHR3* and *TRRAP* (Supplementary Fig. S1). Sequence data have been deposited in NCBI Sequence Read Archive under the accession SRA061758. Other fusion events will be described elsewhere (manuscript in preparation).

### 3.2 Simulation experiment

To get a better understanding of the two modules that form the basis of VFS: the CS and the RP module, we conducted a set of simulation experiments. Our aim was to determine the sequencing depth required to identify a fusion event using either or both methods. Synthesized data allow us to know beforehand where the virus has fused with the host chromosome. The simulation experiment showed that VFS is highly sensitive and accurate. While the RP module reports fusion events with accuracy equal to the inner insertion length, the CS method was able to identify 90% of the fusion events within an accuracy of ±3 bp. Combining the two methods gave the best overall performance. Our simulation also determined that sequencing depth coverage of 10× was sufficient for the detection accuracy to saturate. (Supplementary Section S3).

### 3.3 Re-analysis of real DNA-Seq data

We re-analyzed the whole genome sequencing data of HBV-infected HCC samples using VFS. Two samples (198T and 268T) were randomly chosen (Sung *et al.*, 2012). Remarkably, VFS pinpointed all the exact fusion breakpoints reported by Sung *et al.* Sung reported viral–human integration events only at the genomic DNA level, and it is currently unknown if those reported fusion events would be transcribed. On the other hand, we generated our own RNA-Seq data on one HBV-infected HCC cell line, and then identified and validated three fusion events that are being actively transcribed. In terms of number of reported integration events, Sung *et al.* reported an average of two validated integrations per cell lines, which is comparable with our findings (Supplementary Section S4).

## 4 CONCLUSION

To the best of our knowledge, VFS is the first approach for simultaneously discovering novel viral–human fusion events and reconstructing transcript sequences at single-base resolution. VFS represents an improvement on a methodology that will help with the discovery of viral integration events and expressed transcripts in diseases with viral integration.

## Supplementary Material

Supplementary Data
