# Supplementary material for: ViralFusionSeq: accurately discover viral integration events and reconstruct fusion transcripts at single-base resolution
Source: Bioinformatics. 2013 Jan 12;29(5):649–51. doi: 10.1093/bioinformatics/btt011 (PMC3582262; doi:10.1093/bioinformatics/btt011)
Supplement: Supplementary Data [file supp_29_5_649__index.html]

ViralFusionSeq: accurately discover viral integration events and reconstruct fusion transcripts at single-base resolution — ViralFusionSeq: accurately discover viral integration events and reconstruct fusion transcripts at single-base resolution — Supplementary Data 

# ViralFusionSeq: accurately discover viral integration events and reconstruct fusion transcripts at single-base resolution

## Supplementary Data

files

**Files in this Data Supplement:**

- Supplementary Data - pdf file
