# Supplementary material for: ViralFusionSeq: accurately discover viral integration events and reconstruct fusion transcripts at single-base resolution
Source: Bioinformatics. 2013 Jan 12;29(5):649–51. doi: 10.1093/bioinformatics/btt011 (PMC3582262; doi:10.1093/bioinformatics/btt011)
Supplement: Supplementary Data [file supp_btt011_VFS.supp.pdf]

Supplementary Material for

ViralFusionSeq: Accurately discover viral integration events and reconstruct fusion transcripts in single-base resolution

Jing-Woei Li[1-3], Raymond Wan[1,2], Chi-Shing Yu[1-2], Ngai Na Co[3], Nathalie Wong[3],Ting-Fung Chan[1,2*]

[1]School of Life Sciences, [2]Hong Kong Bioinformatics Center, and [3]Department of Anatomical and Cellular Pathology, The Chinese University of Hong Kong, Shatin, Hong Kong

S1. VIRALFUSIONSEQ COMPARED WITH OTHER TOOLS--3

Human-Human Gene fusion tools --3
Chimera discovery tools ---3
De novo assembler ---3
Viral fusion tool ---4
Virus detection tool --4
Conclusion ---5
Terminology --7

S2. RNA-SEQ EXPERIMENT --8

Cell culture and RNA-Seq --8
Validation of Human-Viral Fusion transcript--8

S3. SEQUENCING DEPTH REQUIRED FOR FUSION EVENT DETECTION IN GENOMIC DNA LEVEL --- 10

Software--- 10
Data --- 12
Workflow --- 12
Results -- 15
Summary of simulation -- 18

S4. VFS PINPOINTS VIRAL-HUMAN BREAKPOINTS IN REAL DNA-SEQ DATASET-------------------- 19

S5. REFERENCES --- 20

Supplementary Table 1: Sequencing summary and statistics 8
Supplementary Table 2: Characteristics of various gene fusion tools. 6
Supplementary Table 3: ViralFusionSeq and VirusSeq compared 7
Supplementary Table 4: In concordance with Sanger sequencing, VFS located all viral-human breakpoints in real whole genome sequencing data 19

Supplementary Figure 1: (A) Validation of HBV-Human fusion event identified by VFS using RT-PCR and Sanger sequencing. (B) Schematic diagram (in proportion) of the mapping result along the fusion transcript...9
Supplementary Figure 2: Overview of the simulation procedure 13
Supplementary Figure 3: Assessment of viral-human breakpoints by the RP method 14
Supplementary Figure 4: Accuracy of viral-human breakpoints against various distance thresholds around the predicted location 15
Supplementary Figure 5: Accuracy of viral-human breakpoints versus sequencing coverage. 17

S1. ViralFusionSeq compared with other tools

Features of numerous tools were compared against ViralFusionSeq (**Supp. Table 1**). Detailed discussion is as follows.

Human-Human Gene fusion tools

Existing gene fusion discovery tools have been developed to find human-human gene fusions which require gene annotation or pre-built transcriptome databases. Specifically, these tools have to construct a putative fusion library followed by splitting unmapped sequences into segments (mostly into arbitrarily defined lengths between 22 to 35bp; deFuse and SASR were the exceptions that employ dynamic programming) and then re-map those segments onto junction libraries. Therefore, these tools are not directly comparable to ViralFusionSeq. Nevertheless, we compared ViralFusionSeq to all these tools and highlighted the uniqueness of VFS. More comprehensive review of fusion tools is described elsewhere (Wang, et al., 2012).

Chimera discovery tools

Viral-fusion sequences are chimeric sequences, consisting of viral and human components. Existing chimera discovery tools are not ideal for viral fusion discovery. Indeed, Chimera Slayer (Haas, et al., 2011), UCHIME (Edgar, et al., 2011) and DECIPHER (Wright, et al., 2012) are the most widely used chimera detection tools. These tools are specifically designed for the removal of chimeric 16S amplicon in metagenomic studies and PCR artifacts in sequencing. It could be debatable that by modifying the reference sequences, these tools might also find viral-human sequences. However, their fundamental designs are limited and are generally not applicable to genome-wide data analysis because: **(1)** only single-end data are supported; (**2**) fusion breakpoints would not be found; and (**3**) these tools split each sequence read into chunks and search against pre-defined sequence databases that are assumed to be free of chimera. Subsequently, any read with portions significantly matched to 2 independent references is taken to be chimeric. At best these tools could only qualitatively assess if the read is likely to be chimera.

De novo assembler

Theoretically, whole transcriptome *de novo* assembly might be able to discover viral-human transcript. We ran one of the state-of-art *de novo* assemblers: Trinity (r2012-10-05) with

HKCI5a RNA data in paired end mode. We parsed the output of Trinity.fasta and compared it to the Sanger validated fusion transcript sequences. Trinity could not assemble the fusion transcript.

Viral fusion tool

To the best of our knowledge, SeqMap 2.0 and VirusSeq are the only two tools that are comparable to ViralFusionSeq that also attempts to find viral integration events. However, SeqMap 2.0 requires the input of pre-defined viral features, and thus cannot discover novel viral components that are involved in fusion, while VirusSeq only finds viral-human fusion events. VirusSeq is a tool for **(1)** detection of the presence of viral species in NGS data, and (2) finding viral integration events. VirusSeq, a recently published tool, uses discordant read pair information combined with clustering to locate regions of a chromosome that fused with a virus.

The most notable difference between ViralFusionSeq (VFS) and VirusSeq is that VFS uses both read pair (RP) and clipped sequence (CS) information to find viral fusion events and breakpoints. Using the latter, VFS is able to discern fusion breakpoints accurately within a few base pairs. While it is possible to use the RP module to determine the fusion breakpoint by aggregating the reads identified by the RP module, a very high coverage of reads would be required. This would be infeasible for RNA-Seq data because expression of those transcripts can be low. Besides, breakpoints resulting from complex fusion events like that of viral-chr7(CDHR7)-TRRAP, as illustrated in **Supp. Figure 1**, would not be detected by the RP module alone. For these reasons, the Clipped Sequence (CS) module is advantageous since it exploits soft-clipping information already present during the alignment stage. Other marked differences are outlined in the **Supp. Table 2**.

Terminology:

Event: An event is a biological phenomenon of sequence integration between human and virus

Detection: Finding the number of reads passing a certain read-count threshold, suggesting the presence of viral species in the NGS dataset.

Virus detection tool

PathSeq and SRSA are software released in 2011 for the discovery of viral identity in NGS datasets. They assume each read either originates from the viral genome or the host

genome. Both SRSA and PathSeq (latest version as of Dec 2012) do not attempt to identity viral integration events nor re-construct viral fusion transcripts.

Conclusion

Taken all these points together, we strongly believe ViralFusionSeq is unique for simultaneously discovering viral-fusion and reconstructing fusion transcript at the genome-wide scale.

Supplementary Table 1: Characteristics of various gene fusion tools. ViralFusionSeq, SeqMap 2.0 and VirusSeq are placed together (green filled-in area) because they are comparable. Virus detection tools are placed under the blue filled-in area. Other tools are sorted in alphabetical order. The human-human gene fusion tools are placed under the grey filled-in area, while the *de novo* assemblers are placed in the violet area.

Tool	DNA-Seq	RNA-Seq	Applicable to viral fusion	Reference based	*de novo* based	Single-end data	Paired-end data	Remarks	References
**ViralFusionSeq**	♦	♦		♦	♦	♦	♦	Leverage both clipped sequence and paired-end evidences to discover novel viral fusion	http://sourceforge.net/projects/viralfusionseq/
**SeqMap 2.0**			♦	♦				Could not discover viral fusion involving novel gene features. Applicable only to 454 data	(Hawkins, et al., 2011)
**VirusSeq**	?	♦		♦			♦	See Supp. Table 3 for detailed comparison between ViralFusionSeq and VirusSeq	(Chen, et al., 2012)
**PathSeq**	♦	♦		♦		♦	♦	Virus detection in NGS dataset only	(Kostic, et al., 2011)
**SRSA**		♦		♦		♦	♦	Virus detection in NGS dataset only	(Isakov, et al., 2011)
**ChimeraScan**				♦				Fusion discovery based on discordant read	(Iyer, et al., 2011)
**Comrad**	♦			♦				Require both DNA-Seq and RNA-Seq dataset	(McPherson, et al., 2011)
**DeFuse**				♦				Paired-end fusion discovery follows by targeted search for split read using dynamic programming	(McPherson, et al., 2011)
**EricScript**				♦				Fusion discovery by paired end read mapped on pre-built transcriptome reference, follows by BLAT recalibration	(Benelli, et al., 2012)
**FusionAnalyzer**				♦				Fusion discovery by paired end read. Construction of exons of genes involved in fusion, follows by mapping of unmapped reads onto these synthetic junctions	(Piazza, et al., 2012)
**FusionFinder**				♦				Partitioned unmapped reads into segments of 40% (default) to 50% of original read and remap	(Francis, et al., 2012)
**FusionHunter**				♦				Partitioned of unmapped reads into fixed length (<50% of the read). Requires gene annotation	(Li, et al., 2011)
**FusionMap**	♦			♦		♦		Fusion discovery depends on construction of pseudo fusion sequences library	(Ge, et al., 2011)
**FusionSeq**		♦		♦			♦	Fusion discovery based on discordant read	(Sboner, et al., 2010)
**SASR**				♦		♦		Consider spliced-read and paired-end alignment independently	(Sakarya, et al., 2012)
**ShortFuse**				♦				Partitioned unmapped reads into segments of fixed length (22bp) during split-read analysis	(Kinsella, et al., 2011)
**SnowShoes-FTD**				♦				Partitioned unmapped reads into segments of fixed length (32bp) during split-read analysis	(Asmann, et al., 2011)
**SOAPfusion**				♦				No technical information is available	http://soap.genomics.org.cn/SOAPfusion.html
**SPALN2**				♦				Requires transcript database for fusion discovery	(Iwata and Gotoh, 2012)
**TopHat-Fusion**				♦		♦		Partitioned unmapped reads into 3 segments of defined length during split-read analysis	(Kim and Salzberg, 2011)
**Trans-ABySS**		♦		*de novo* assembly			♦	Not designed for viral fusion	(Robertson, et al., 2010)
**Trinity**				*de novo* assembly		♦		Not designed for viral fusion	(Grabherr, et al., 2011)

Supplementary Table 2: ViralFusionSeq and VirusSeq compared

ViralFusionSeq	VirusSeq
N/A	Viral detection in NGS dataset against a viral database using read-count method
Discovery of viral integration event	Discovery of viral integration event
Determination of viral-human ***breakpoint*** at base-pair resolution	N/A
Re-construction of viral fusion *transcript*	N/A
Support single end sequence data	N/A
Discover complex fusion transcripts result from viral-human and human-human fusion events	N/A
Method tested on both RNA-Seq and DNA-Seq data	Method tested on RNA-Seq data only
Sensitivity and specificity defined by whether VFS finds the actual viral-human *breakpoint* & fusion event for Clipped Sequence and Read Pair module, respectively	Sensitivity and specificity defined by ***detection*** of virus among all reads

Terminology

Event: An event is a biological phenomenon of sequence integration between human and virus.

Detection: Finding the number of reads passing a certain read-count threshold, suggesting the presence of viral species in the NGS dataset.

S2. RNA-Seq experiment

Cell culture and RNA-Seq

The method for the cell culture of HKCI-5a, a cell line derived from chronic Hepatitis B (HBV) infected primary hepatocellular carcinoma, was previously described (Chan, et al., 2006).

Sequencing was done by Macrogen (http://www.macrogen.com/), which is affiliated with the Illumina Genome Network. Briefly, the sequencing library was prepared using the Illumina TruSeq RNA Sample Preparation Kit. RNA-Seq was performed using HiSeq 2000, generating paired-end sequence reads of read-length 101bp. Insert size was 350bp. Sequencing statistics is shown at **Supp. Table 3**.

Validation of Human-Viral Fusion transcript

Total RNA was extracted by lysing HKCI-5a cells with TRIzol (Invitrogen, Carlsbad, CA) and treated with RQ1 RNase-free DNase (Promega Corporation, Madison, WI) to eliminate genomic DNA carryover. First strand cDNA was synthesized from 0.5 ug total RNA by MultiScribe reverse transcriptase (Applied Biosystems) using random hexanucleotide primers and according to the manufacturer's instructions. 20 ng of the synthesized cDNA was used to perform the amplification of HBV-TRRAP fusion transcript using AmpliTaq Gold® 360 PCR Master Mix (Applied Biosystems). Primers for HBV-TRRAP fusion transcript were 5'-CTTTGGAGCATGGACATTGA-3' (sense) and 5'-CAGACAGTGGATTCCCAGGT-3' (anti-sense). After 40 cycles of the following parameters: 30 sec at 95°C, 30 sec at 50°C, followed by 30 sec at 72°C, PCR products were resolved in a 1.5% agarose gel and sequenced by Sanger sequencing. Gel photo, Sanger flowgram and schematic diagram of reads mapping on the fusion transcript is shown in **Supp. Figure 1**.

Supplementary Table 3: Sequencing summary and statistics

Seq ID	Description	#Raw reads (million)	Raw throughput (Gigabases)	#Trimmed reads (million)	Trimmed throughput (Gigabases)
**HKCI-5a**	HBV infected cell line	111.1	11.2	108.2	10.6

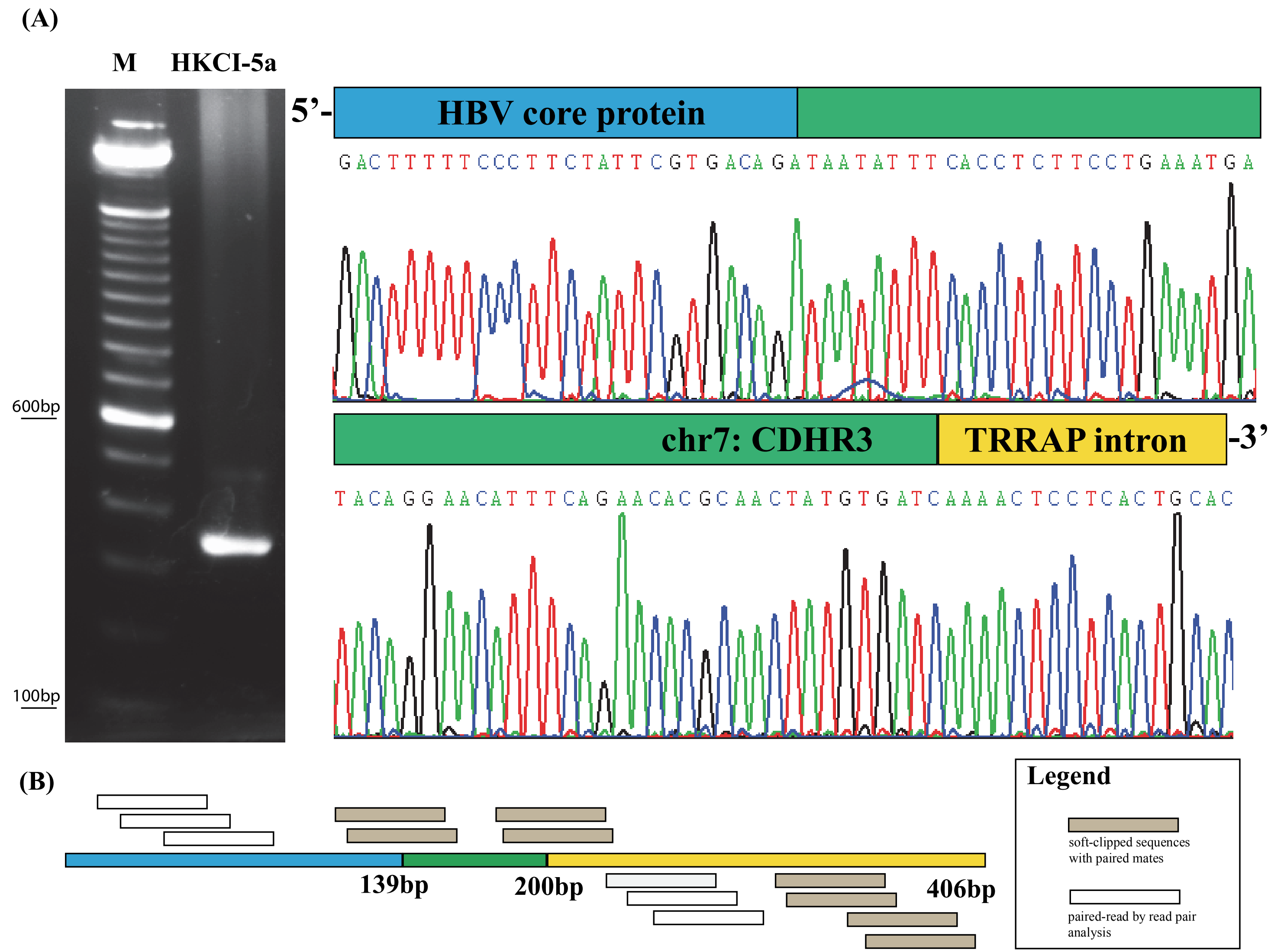

Supplementary Figure 1: (A) Validation of HBV-Human fusion event identified by VFS using RT-PCR and Sanger sequencing. **(B)** Schematic diagram (in proportion) of the mapping result along the fusion transcript. The fusion event was re-constructed by VFS using targeted *de novo* assembly using (**1**) soft-clipped sequences and their paired reads, (**2**) reads from Read Pair analysis and (**3**) reads mapped into the vicinity (500bp) of the human features reported in read-pair analysis

S3. Sequencing depth required for fusion event detection at genomic DNA level

The Lander-Waterman statistics dictate that genomic coverage follows a Poisson distribution (Lander and Waterman, 1988). We reasoned that a combined factor of sequencing coverage, analysis tool's sensitivity and the percentage of viral infected cells among the entire population (which might be low) would affect the discovery of viral integration events.

While the percentage of viral infected cells depends on samples, we hope to address the sequencing coverage and the sensitivity issue. The purpose of the simulation is to determine what is the sequencing coverage needed to discover all viral integration events. At the same time we are also asking whether or not VFS can locate known fusion breakpoints. And if yes, how sensitive is VFS.

In order to get a better understanding of the two modules that form the basis of VFS – the Clipped Sequence (CS) and the Read Pair (RP) module – we conducted a set of simulation experiments. Our aim is to determine the sequencing depth required to identify a fusion event using either or both modules. Synthesized data allows us to know beforehand where the virus has fused with the host chromosome. The following describes the software, data, overall workflow, results, and summary of this simulation experiment.

Software

The simulation requires Perl scripts and one publicly available software tool. The Perl scripts created for this simulation are also included as part of the VFS software distribution. The two main scripts are *simulate-viralfusion.pl* and *evaluate-viralfusion.pl*. The first is used for introducing parts of the viral genome into the host chromosome at random locations. The second takes the outputs from the two VFS modules and the true fusion locations and reports the overall accuracy.

The software required is the Wgsim software (a tool for simulating sequence reads from a reference genome) by Heng Li, whose source is available from GitHub at https://github.com/lh3/wgsim/. This program is based on the simulator for the MAQ short read

mapper (Li, et al., 2008) and its purpose is to extract paired-end reads at random from a genome. Version 0.3.1-r13 of the software was employed.

Both of the Perl scripts include in-line documentation using Perldoc to explain the various parameters available to the user. In addition to the two genomes, *simulate-viralfusion.pl* requires:

1. the number of insertions and
2. a range indicating what percentage of the virus to insert.

Based on these parameters, the script uses a uniform distribution to (**1**) select how much of the virus to take for each fusion event and (**2**) where to insert the virus. The script assumes a 100% infection rate by the virus on the supplied genome.

The parameters employed for Wgsim are described in later sections. The following parameters were left as default:

- base error rate = 0.020
- outer distance between the two ends = 500
- standard deviation = 50
- rate of mutations = 0.0010
- fraction of indels = 0.15
- probability an indel is extended = 0.30

Data

Experiments were performed using an Ubuntu 12.04 system with an Intel (TM) Xeon CPU E7-4860 (2.27 GHz) with 24 GB cache and 512 GB RAM. However, only a small portion (< 16 GB) of memory was ever used by VFS. Perl v5.14.2 was used to run VFS.

Kraus *et. al.* has reported that HPV integration sites were found randomly distributed throughout the genome (Kraus, et al., 2008). Wentzensen *et. al.* also reported that "HPV integration loci were distributed over almost all human chromosomes …… no preferential site of integration motif could be identified." (Wentzensen, et al., 2002). Indeed, some virus (e.g. HIV) may exhibit distinct integration sites preference for certain genomic features (e.g. transcription start site, CpG islands, etc) than other virus (e.g. ASLV, MLV). However, in the context of human chromosome as a whole, integration events were observed quite evenly across all chromosomes (Mitchell, et al., 2004).

The virus we selected for our simulation experiments was HIV-1. Since we could not find any strong evidence that any chromosome was more susceptible to HIV-1 than any other chromosome, we selected chromosome 1 for the basis of our simulation experiment. Our main reason for this choice is that chromosome 1 is the largest human chromosome with high sequence complexity. Using only chromosome 1 allowed us to perform simulations of insertion of enough viral segments to assess the accuracy of VFS using an extremely high fold of simulated sequencing coverage.

We obtained chromosome 1 of hg19 retrieved from UCSC and the Human immunodeficiency virus 1 from NCBI (Accession: NC_001802.1). The sizes of both are 249,250,621 and 9,181 bps, respectively.

Workflow

Our simulation procedure is summarized in **Figure S2**. It can be divided roughly into 4 stages. The first stage introduces viral fusions into the genome. The second stage generates paired-end reads with mutations from this infected chromosome. The third step runs the VFS pipeline using

this data. Finally, the last step performs the evaluation and outputs an overall accuracy for the simulation. We elaborate on each of these steps below.

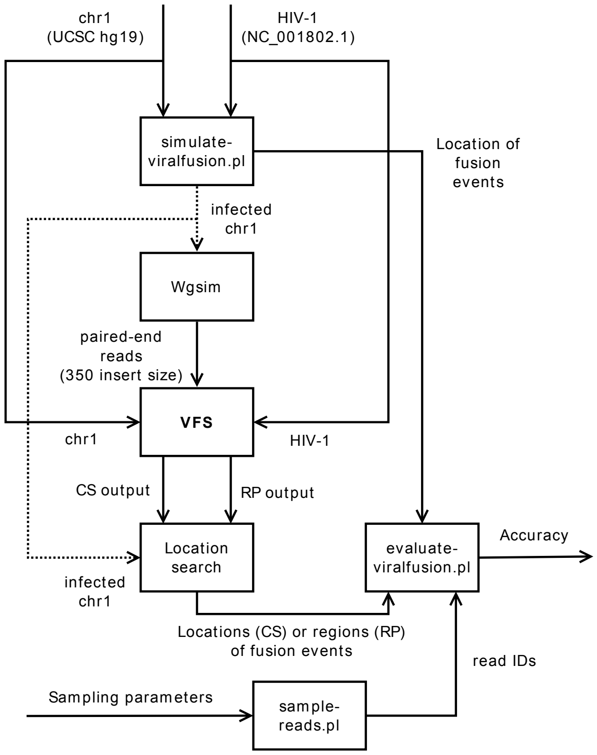

Supplementary Figure 2: Overview of the simulation procedure

First, viral sequences are introduced at random into the host chromosome using *simulate-viralfusion.pl*. The viral genome is divided into non-redundant segments of 400 nucleotides and then 75-100% of each segment is sampled to yield an insertion segment. This translates into 22 segments with lengths of 300-400 bps each for insertion. Since there were 22 insertions, there are 44 fusion breakpoints to predict (the positions before and after the virus segment in the infected chromosome's genome). The script was run with these options: "--virus-block-len 400 --low-virus 75 --high-virus 100".

Second, we extracted 250,000,000 paired-end reads from this simulated chromosome using *Wgsim*. Each read was 101 bps in length. This gives coverage of about 202x. All other

parameters for *Wgsim* were left as default, as explained earlier. Thus, *Wgsim* was run with these options: "-1 101 -2 101 -N 250000000 -d 350".

Third, these inputs are used by the VFS pipeline to produces two outputs, one for the Clipped Sequence (CS) module and the other for the Read Pair (RP) module. Both consist of a set of sequences. As coordinates of these sequences are required relative to the infected chromosome 1, an additional location search for this simulation was performed separately. The result is (**1**) a set of base-level locations for the CS module and (**2**) a set of regions of fusion events for the RP module.

Finally, together with the true locations of the fusion events (one of the outputs of *simulate-viralfusion.pl*), the *evaluate-viralfusion.pl* script determines the accuracy of VFS. In the case of the CS module, an accurate match means we predicted the location of the fusion exactly. But for the RP module, since its accuracy is equal to the insertion distance between the two reads, we accept predictions that fall within the region bounded by the last position of the forward read and the first position of the reverse read.

With an insertion size of 350 bp and pairs of reads of 101 nucleotides each, the allowable error is ±148 bp counting inward, as depicted in **Figure S3**.

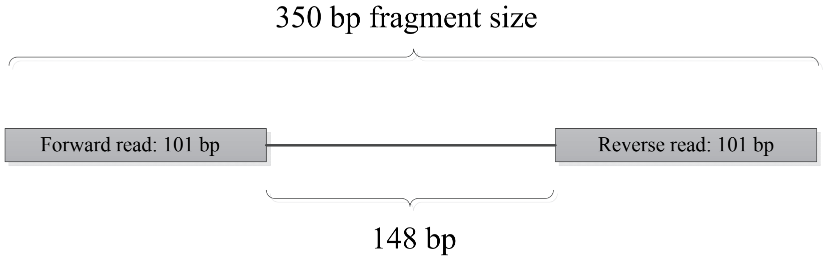

Supplementary Figure 3: Assessment of viral-human breakpoints by the RP module

At the bottom of the workflow of **Figure S2** is a third Perl script for sampling. This script, *sample-reads.pl*, is also included as part of the VFS distribution. Its purpose is explained below.

Results

As a starting point, we investigate the accuracy of the CS module in predicting the exact location of a fusion event, in the presence of both simulated mutations and sequencing errors in the reads. Recall that the CS module is able to report breakpoints at the base-level due to its use of clipped sequences.

Here, "accuracy" refers to the percentage of viral fusion breakpoints that were detected out of the 44 that were introduced into the UCSC hg19 chromosome 1. We used window size as an evaluation parameter and consider a breakpoint to be correctly detected if the true site is within the window around the predicted location. Thus, its accuracy will increase with larger window sizes. Our findings are shown in **Figure S4**.

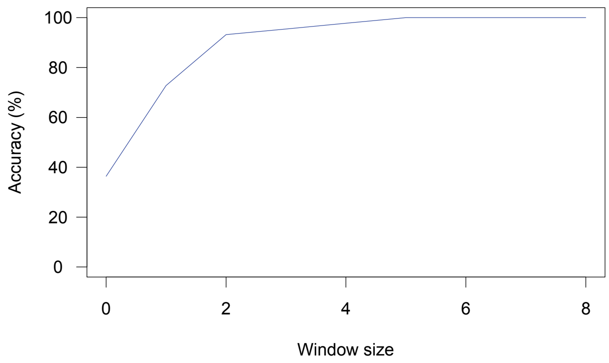

Supplementary Figure 4: Accuracy of viral-human breakpoints against various distance thresholds around the predicted location

These results show that 40% of the 44 breakpoints can be predicted exactly. If we allow just ±1 bp or ±2 bps, then accuracy goes up to around 70% and 90%, respectively. When the window size is up to ±5 bps, then all breakpoints have been accounted for. In the next set of experiments, we use window sizes of 0 bp and ±3 bps in order to represent two different extremes.

While one aim of this simulation is to evaluate VFS, the large coverage of simulated reads also allows us to report on the effectiveness of viral fusion discovery as a function of read coverage. We can achieve this by randomly selecting reads from the 250,000,000 pairs of reads and then reporting on the average accuracy of the CS and RP modules. Random sampling was done using the *sample-reads.pl* script.

VFS was ran only once with all 250,000,000 pairs of reads. This is because random sampling does not require us to re-run VFS. Instead, we only have to take note of how many fusion breakpoints can still be detected. This was repeated 100 times to yield error bars indicating one standard deviation.

Our results for these simulations with random sampling of reads are presented in the two panels of Figure S4. Panel (a) uses a window size of 0 bp; the panel on the right (b) uses a window size of ±3 bps. Note that the RP module is unaffected by window size. Each panel plots accuracy versus coverage, where accuracy is the percentage of the 44 fusion points that were correctly detected. The three lines in each panel represent (1) CS and RP module, (2) RP module only, and (3) CS module only.

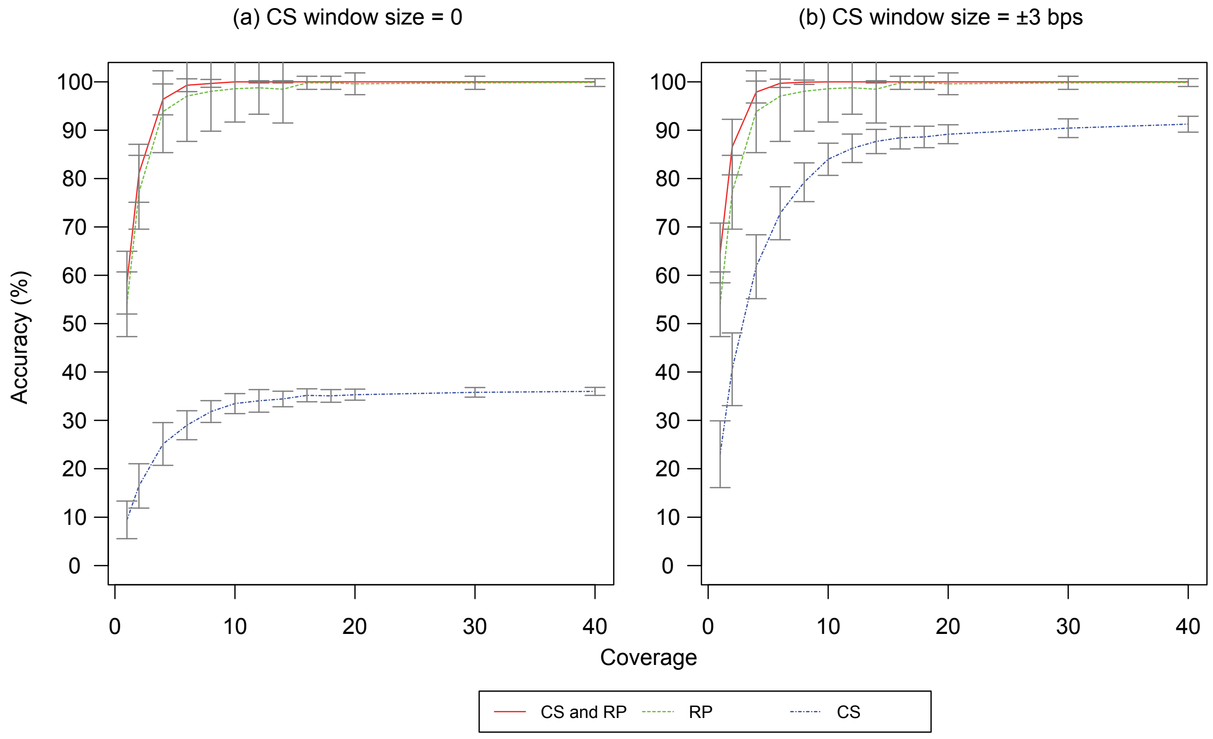

Supplementary Figure 5: Accuracy of viral-human breakpoints versus sequencing coverage. (**a**) Distance threshold of 0 bp was used; (**b**) Distance threshold of ±3 bp was used. Abbreviation: RP: Read Pair analysis; CS: Clipped Sequence information

As **Figure S5** shows, accuracy steadily increases for all modules while the standard deviation is particularly high at low coverages. At a coverage of 15x, accuracy begins to level out. Thus, it would appear that 202x coverage is unnecessary for predicting viral breakpoints.

With a window size of 0 bp, 40% of the breakpoints were reported. As expected, the CS module improves significantly and found around 95% of the total viral fusion breakpoints if we relax the evaluation criteria to allow a larger window around the predicted position (±3 bp). While the RP module performs very well, we note that the RP module is imprecise since it can only indicate that a viral fusion breakpoint exists in a region equal to the size of the inner insertion size.

Summary of simulation

The simulation has shown that VFS is highly sensitive and accurate. The CS module can be improved noticeably through a slightly larger window of a few base pairs. This is in contrast to the RP module which gives a resolution equal to the inner insertion size (i.e., the distance between the two read pairs).

We note that an alternative simulation framework was used by PathSeq (Kostic, et al., 2011). They assumed that each sequence read either entirely originates from the viral or the host genome, but not a combination of the two. Thus, their software resembles the RP module. In contrast, we have inserted the viral genome *first* before randomly extracting reads. We believe this approach is more realistic and is what the CS module was designed for. Combining both the CS and RP modules takes into account the two different scenarios.

These simulation results have demonstrated that sequencing depth as low as 10x may be sufficient for detecting breakpoints. Obviously, this depends on various factors, including the similarity between the virus genome and the host genome and how much of the viral genome is inserted.

S4. VFS pinpoints viral-human breakpoints in real DNA-Seq dataset

We randomly chose 2 cell lines from one recent paper on HBV fusion and re-analyzed the DNA-Seq data by VFS (Sung, et al., 2012). Data was retrieved from http://gigadb.org/hepatocellular-carcinoma/. VFS was run with default parameters. Potential PCR duplicates, where the 5' mapping position was the same, were discarded. VFS exploited clipped-sequence information and could locate all the viral-human breakpoints (**Table S4**). These breakpoints were previously confirmed by Sung *et. al.* using Sanger sequencing.

For each event, Sung *et. al.* reported the number of supporting clusters instead of the number of sequence reads in supplementary materials. The exact clustering procedure and the number of reads that make up the clusters were not mentioned. We provided the exact number of reads supporting the fusions. In our opinion, these 2 sets of columns (columns 4-5 and column 7 of Table S4) are not directly comparable.

Supplementary Table 4: In concordance with Sanger sequencing, VFS located all viral-human breakpoints in real whole genome sequencing data

Sample	Gene	Breakpoint found by clipped-sequence information	VFS # Clipped Seq evidences	VFS # Read pair evidences	Exact hg19 breakpoint found by Sanger sequencing	#Cluster support
198T	hTERT	chr5:1269387	3	29	chr5:1269387	13
	hTERT	chr5:1269405	12		chr5:1269405	12
268T	hTERT	chr5:1292391	5	29	chr5:1292391	8
	hTERT	chr5:1292403	5		chr5:1292403	9
	CCNE1	chr19:30298787	16	80	chr19:30298787	3

S5. References

ASMANN, Y.W., *ET AL.* (2011) A NOVEL BIOINFORMATICS PIPELINE FOR IDENTIFICATION AND CHARACTERIZATION OF FUSION TRANSCRIPTS IN BREAST CANCER AND NORMAL CELL LINES, *NUCLEIC ACIDS RES*, **39**, E100.

BENELLI, M., *ET AL.* (2012) DISCOVERING CHIMERIC TRANSCRIPTS IN PAIRED-END RNA-SEQ DATA BY USING ERICSCRIPT, *BIOINFORMATICS*.

CHAN, K.Y., *ET AL.* (2006) POSITIONAL EXPRESSION PROFILING INDICATES CANDIDATE GENES IN DELETION HOTSPOTS OF HEPATOCELLULAR CARCINOMA, *MOD PATHOL*, **19**, 1546-1554.

CHEN, Y., *ET AL.* (2012) VIRUSSEQ: SOFTWARE TO IDENTIFY VIRUSES AND THEIR INTEGRATION SITES USING NEXTGENERATION SEQUENCING OF HUMAN CANCER TISSUE, *BIOINFORMATICS*.

EDGAR, R.C., *ET AL.* (2011) UCHIME IMPROVES SENSITIVITY AND SPEED OF CHIMERA DETECTION, *BIOINFORMATICS*, **27**, 2194-2200.

FRANCIS, R.W., *ET AL.* (2012) FUSIONFINDER: A SOFTWARE TOOL TO IDENTIFY EXPRESSED GENE FUSION CANDIDATES FROM RNA-SEQ DATA, *PLOS ONE*, **7**, E39987.

GE, H., *ET AL.* (2011) FUSIONMAP: DETECTING FUSION GENES FROM NEXT-GENERATION SEQUENCING DATA AT BASE-PAIR RESOLUTION, *BIOINFORMATICS*, **27**, 1922-1928.

GRABHERR, M.G., *ET AL.* (2011) FULL-LENGTH TRANSCRIPTOME ASSEMBLY FROM RNA-SEQ DATA WITHOUT A REFERENCE GENOME, *NAT BIOTECHNOL*, **29**, 644-652.

HAAS, B.J., *ET AL.* (2011) CHIMERIC 16S RRNA SEQUENCE FORMATION AND DETECTION IN SANGER AND 454-PYROSEQUENCED PCR AMPLICONS, *GENOME RES*, **21**, 494-504.

HAWKINS, T.B., *ET AL.* (2011) IDENTIFYING VIRAL INTEGRATION SITES USING SEQMAP 2.0, *BIOINFORMATICS*, **27**, 720-722.

ISAKOV, O., MODAI, S. AND SHOMRON, N. (2011) PATHOGEN DETECTION USING SHORT-RNA DEEP SEQUENCING SUBTRACTION AND ASSEMBLY, *BIOINFORMATICS*, **27**, 2027-2030.

IWATA, H. AND GOTOH, O. (2012) BENCHMARKING SPLICED ALIGNMENT PROGRAMS INCLUDING SPALN2, AN EXTENDED VERSION OF SPALN THAT INCORPORATES ADDITIONAL SPECIES-SPECIFIC FEATURES, *NUCLEIC ACIDS RES*, **40**, E161.

IYER, M.K., CHINNAIYAN, A.M. AND MAHER, C.A. (2011) CHIMERASCAN: A TOOL FOR IDENTIFYING CHIMERIC TRANSCRIPTION IN SEQUENCING DATA, *BIOINFORMATICS*, **27**, 2903-2904.

KIM, D. AND SALZBERG, S.L. (2011) TOPHAT-FUSION: AN ALGORITHM FOR DISCOVERY OF NOVEL FUSION TRANSCRIPTS, *GENOME BIOL*, **12**, R72.

KINSELLA, M., *ET AL.* (2011) SENSITIVE GENE FUSION DETECTION USING AMBIGUOUSLY MAPPING RNA-SEQ READ PAIRS, *BIOINFORMATICS*, **27**, 1068-1075.

KOSTIC, A.D., *ET AL.* (2011) PATHSEQ: SOFTWARE TO IDENTIFY OR DISCOVER MICROBES BY DEEP SEQUENCING OF HUMAN TISSUE, *NAT BIOTECHNOL*, **29**, 393-396.

KRAUS, I., *ET AL.* (2008) THE MAJORITY OF VIRAL-CELLULAR FUSION TRANSCRIPTS IN CERVICAL CARCINOMAS COTRANSCRIBE CELLULAR SEQUENCES OF KNOWN OR PREDICTED GENES, *CANCER RES*, **68**, 2514-2522.

LANDER, E.S. AND WATERMAN, M.S. (1988) GENOMIC MAPPING BY FINGERPRINTING RANDOM CLONES: A MATHEMATICAL ANALYSIS, *GENOMICS*, **2**, 231-239.

LI, H., RUAN, J. AND DURBIN, R. (2008) MAPPING SHORT DNA SEQUENCING READS AND CALLING VARIANTS USING MAPPING QUALITY SCORES, *GENOME RES*, **18**, 1851-1858.

LI, Y., *ET AL.* (2011) FUSIONHUNTER: IDENTIFYING FUSION TRANSCRIPTS IN CANCER USING PAIRED-END RNA-SEQ, *BIOINFORMATICS*, **27**, 1708-1710.

MCPHERSON, A., *ET AL.* (2011) DEFUSE: AN ALGORITHM FOR GENE FUSION DISCOVERY IN TUMOR RNA-SEQ DATA, *PLOS COMPUT BIOL*, **7**, E1001138.

McPherson, A., *et al.* (2011) Comrad: detection of expressed rearrangements by integrated analysis of RNA-Seq and low coverage genome sequence data, *Bioinformatics*, **27**, 1481-1488.
Mitchell, R.S., *et al.* (2004) Retroviral DNA integration: ASLV, HIV, and MLV show distinct target site preferences, *PLoS Biol*, **2**, E234.
Piazza, R., *et al.* (2012) FusionAnalyser: a new graphical, event-driven tool for fusion rearrangements discovery, *Nucleic Acids Res*, **40**, e123.
Robertson, G., *et al.* (2010) De novo assembly and analysis of RNA-seq data, *Nat Methods*, **7**, 909-912.
Sakarya, O., *et al.* (2012) RNA-Seq mapping and detection of gene fusions with a suffix array algorithm, *PLoS Comput Biol*, **8**, e1002464.
Sboner, A., *et al.* (2010) FusionSeq: a modular framework for finding gene fusions by analyzing paired-end RNA-sequencing data, *Genome Biol*, **11**, R104.
Sung, W.K., *et al.* (2012) Genome-wide survey of recurrent HBV integration in hepatocellular carcinoma, *Nat Genet*, **44**, 765-769.
Wang, Q., *et al.* (2012) Application of next generation sequencing to human gene fusion detection: computational tools, features and perspectives, *Brief Bioinform*.
Wentzensen, N., *et al.* (2002) Characterization of viral-cellular fusion transcripts in a large series of HPV16 and 18 positive anogenital lesions, *Oncogene*, **21**, 419-426.
Wright, E.S., Yilmaz, L.S. and Noguera, D.R. (2012) DECIPHER, a search-based approach to chimera identification for 16S rRNA sequences, *Appl Environ Microbiol*, **78**, 717-725.
